# Printing 3D models of canine jaw fractures for teaching undergraduate veterinary medicine^1^

**DOI:** 10.1590/s0102-865020190090000006

**Published:** 2019-12-05

**Authors:** Agnes de Souza Lima, Marcello Machado, Rita de Cassia Ribeiro Pereira, Yuri Karaccas de Carvalho

**Affiliations:** I M.Sc., Postgraduate Program in Health and Animal Production, Universidade Federal do Acre (UFAC), Rio Branco-AC, Brazil. Acquisition, analysis and interpretation of data; manuscript preparation and writing.; II D.Sc., Department of Anatomy, Universidade Federal do Paraná (UFPR), Curitiba-PR, Brazil. Scientific and intellectual content of the study.; III M.Sc., Health and Sports Science Center, UFAC, Rio Branco-AC, Brazil. Technical procedures.; IV D.Sc., Biological and Natural Sciences Center, UFAC, Rio Branco-AC, Brazil. Manuscript writing, critical revision, final approval.

**Keywords:** Anatomy, Veterinary, Mandibular Fractures, Printing, Three-dimensional, Teaching Materials

## Abstract

**Purpose:**

To develop 3D anatomical models, and corresponding radiographs, of canine jaw fractures.

**Methods:**

A base model was generated from a mandibular bone scan. With this model it was possible to perform fracture planning according to the anatomical location.

**Results:**

The 3D base model of the canine mandible was similar in conformation to the natural bone, demonstrating structures such as canine tooth crowns, premolars and molars, mental foramina, body of the mandible, ramus of the mandible, masseteric fossa, the coronoid process, condylar process, and angular process. It was not possible to obtain detail of the crown of the incisor teeth, mandibular symphysis, and the medullary channel. Production of the 3D CJF model took 10.6 h, used 150.1 g of filament (ABS) and cost US$5.83.

**Conclusion:**

The 3D canine jaw fractures models, which reproduced natural canine jaw fractures, and their respective radiographic images, are a possible source of educational material for the teaching of veterinary medicine.

## Introduction

Mandibular fractures are commonly observed in the clinical routine of small animals and account for approximately 3% of all reported fractures in dogs^1^.

The precise diagnosis of a mandibular fracture determines the appropriate treatment and prognosis of this condition. Considering this, radiographic examination is essential for identifying the exact location and extension of the fracture, enabling appropriate therapeutic planning^2^.

Undergraduate veterinary medicine fracture studies take place through books, medical images and, when there is opportunity, the follow-up of clinical cases. Despite the frequency in the clinical routine, this condition is largely overlooked during the course of study^3^. This fact may be directly related to the absence of clinical cases during study, or the limitation of teaching materials that can fully demonstrate all the aspects this condition presents^4^.

The use of 3D printing in general veterinary medicine is already a reality. There have been great advances in the application of this technology in several areas, such as orthopedics^5^, ophthalmology^6^, diagnostic imaging^7^, treatment of large animals^8,9^, surgical planning^10,11^, and especially in the teaching of veterinary medicine^12^. In the latter, research has concentrated particularly on anatomy^13^ and surgery^14^.

Although there have been several 3D models created that focus on teaching and research, we have not found any reported attempts to reproduce canine jaw fractures (CJF) in veterinary medicine. Thus, this study aimed to produce 3D educational models of CJF.

## Methods

Experimental protocol was approved by the Animal Research Ethics Committee of Universidade Federal do Acre (CEUA-UFAC), protocol number 47/18.

**Figure 1:**
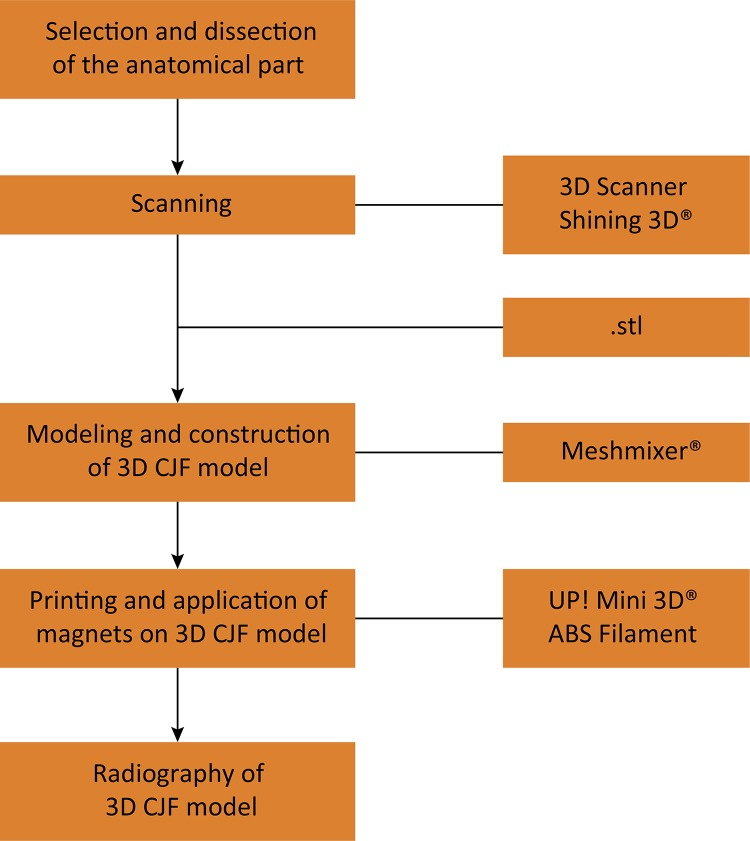
- 3D canine jaw fractures model creation flowchart.

Cadaver jaw from a healthy dog that died of natural causes was macerated and prepared for further scanning manipulation and printing. Radiography of the 3D CJF models was taken and models were generated from the mandibular bone scan. The creation of the 3D CJF model was based on the bone scan, posterior virtual modeling and 3D printing (Fig. 1).

Scanning of the mandibular bone was performed using a 3D Scanner, Model EinScan-SP (Shining 3D^®^, Zhejiang, China), using the EinScan-SP Version 2.6.0.8 software included with the equipment.

Images were saved in .stl format and stored in a database. Subsequently, they were transferred to a 3D creation and manipulation system, Autodesk Meshmixer^®^, version 3.1 (Autodesk Inc.^©^, California, United States), for modeling and composition of the 3D CJF model.

The modeling stage consisted of separating the anatomical regions in which fractures occur. There was no loss of information during this process, and all structures of the mandible were maintained.

Fracture locations reproduced in the 3D CJF models were based on those reported by^15-17^ (Chart 1).

**Chart 1:** Classification of canine jaw fractures (CJF) by location.

Classification	Location
A	Mandibular symphysis
B	Mandibular parasymphysis
C	Canine
D	Premolar
E	Molar
F	Coronoid process
G	Condilar process
H	Ramus of mandible
I	Angular process

Adapted from^15-17^

The constituent parts of each 3D CJF model were printed using UP 3D Mini^®^ (Beijing Tiertime Technology Co. Ltd., Beijing, China), which uses fine quality Fused Deposition Modeling (FDM) technology and ABS-grade thermoplastic material, with a 99% internal fill and a layer thickness of 0.2 mm. After printing, manual finishing was performed. Neodymium magnets, 5 mm in diameter and 2 mm in height, were inserted in the fracture lines of each segment of the model, to enable assembly and disassembly of the parts.

### Radiographic study of 3D CJF models

Four 3D CJFs were created to represent the nine CJF types, according to anatomical location, organized as follows: I - canine/coronoid process, II - parasymphysis/angular process/premolar, III - ramus of the mandible/molar, IV - mandibular symphysis/condylar process.

Representation of the different types of fractures in the 3D CJF model, and their respective radiographic positions, are shown in Chart 2.

**Chart 2:** Canine jaw fractures by model and radiographic positioning.

3D CJF model	Fractures	Positioning
I	Canine	Dorsoventral
	Coronoid process	Laterolateral
II	Parasymphysis	Dorsoventral
	Angular process	Laterolateral
	Premolar	Laterolateral
III	Ramus of the mandible	Laterolateral
	Molar	Laterolateral
IV	Mandibular symphysis	Dorsoventral
	Condylar process	Dorsoventral

After completion of the construction phase, models were taken to the Diagnostic Imaging Center to undergo radiography. 3D CJF models were X-rayed using General Electric model DR-F (General Electric Company^©^, USA) digital X-ray equipment, with a radiation intensity of 40 kilovolt kV and exposure time of 2.51 mAs.

Fracture X-rays were focused on at least one of the positions recommended by Fossum^18^, and Han and Hurd^19^ for mandible radiography. Images were edited using RadiAnt DICOM Viewer^®^ 2009-2017 software (Medixant^®^, Poznan, Poland).

For radiography it was necessary remove the neodymium magnets. Therefore, we secured the fracture foci with high-abrasion transparent double-sided adhesive tape.

## Results

**Figure 2:**
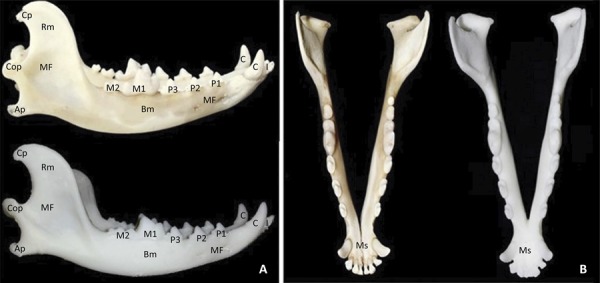
- Canine mandible and corresponding 3D base model, (A) right side view and (B) dorsal view. I. incisor teeth; C. canine teeth; MF. mental foramina; P1. first premolar tooth; P2. second premolar tooth; P3. third premolar tooth; M1. First molar tooth; M2. second molar tooth; Bm. body of the mandible; Cp. coronoid process; Rm. ramus of the mandible; MF. masseteric fossa; Cop. condylar process; Ap. angular process; Ms. mandibular symphysis.

The 3D base model of the mandible showed a similar conformation as the natural bone, maintaining the same length and width, in addition to reproducing the structures that identify the bone. The following anatomical structures were observed: canine tooth crowns, premolar and molar teeth, mental foramina, body of the mandible, ramus of the mandible, masseteric fossa, and coronoid, condylar and angular processes. However, it was not possible to obtain details of the incisor tooth crowns, union of the mandibular symphysis or projection of the mandibular canal (Fig. 2).

After production of all models, the creation time, printing time, amount of material used, and associated costs, were estimated. This information is shown in Table 1, which allows an appreciation of the cost of each model, and the total cost of the 3D CJF model.

**Table 1:** Creation time, print time, quantity of material used, and costs of the 3D CJF.

3D CJF	CREATION TIME (h)	PRINT TIME (h)	MATERIAL USED (g)	COST (US$)
I- Canine/ Coronoid process	0.5	2.7	38.3	1.49
II- Parasymphysis/ Angular process / Premolar	1.0	3.1	41.2	1.60
III- Ramus of the mandible/ Molar	1.0	2.4	36.1	1.40
IV- Mandibular symphysis/Condylar process	0.5	2.4	34.5	1.34
TOTAL (4 x 3D CJF)	3.0	10.6	150.1	5.83

(h) - Hours; (g) - Grams; (US$) – US Dollars; Reference US$ 1.00 = R$3.80; Filament US$30.00

Creation time refers to procedures carried out post-scanning, i.e., the computer based manipulation of images, since the scanning time duration was only 5 min.

Factors that influenced printing time were the number of fractures per model, arrangement of the parts on the printing table, and setting up the printer (internal part fill, print speed, and layer thickness).

Printing costs accounted for the amount of filament used, depreciation of the machine and power consumption. However, the costs of the equipment (3D printer and 3D scanner) were not accounted for.

**Figure 3:**
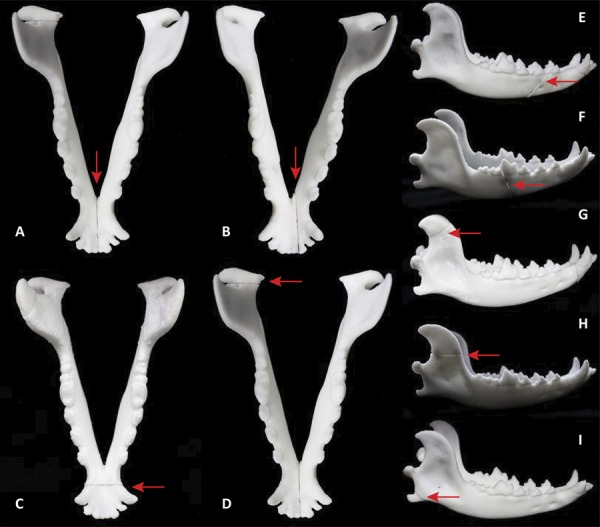
- 3D anatomical models of canine jaw fractures. A. mandibular symphysis; B. mandibular parasymphysis; C. canine region; D. condylar process; E. premolar region; F. molar region; G. coronoid process; H. ramus of the mandible; I. angular process. Arrows indicate fracture foci.

Different types of mandibular fractures were represented by four models. Three of the models represented two types of fracture and one model represented three types of fracture, thus representing nine types of CJF. The combining of multiple fractures within a model was based on attempting to reduce costs. For all models, we aimed to reproduce a fracture in the rostral portion and another in the flow (Fig. 3).

For model I (canine/coronoid process), two cuts were made. In the region of the coronoid process, it was necessary to slightly increase the thickness, to allow the magnet to be fitted, since the original was very thin and incompatible with the width of the joint mechanism (magnet).

For model II (parasymphysis/angular process/premolar), three cuts were made. No adjustments to the parts were necessary, since they all had surfaces compatible with the magnets. The cut representing the fracture in the premolar region was made transversely, resulting in a fracture that is unfavorable to the chewing muscles.

For model III (mandible/molar ramus), two cuts were made, which presented suitable surfaces for the placement of the magnets. For the fracture of the mandibular ramus, a horizontal cut was made, and the cut representing the fracture of the molar region was transverse, corresponding to type of fracture favorable to the chewing muscles.

For model IV (mandibular symphysis/condylar process) two cuts were made. The cut in the mandibular symphysis region was between the intermandibular joint and the incisor teeth. The cut representing the fracture of the condylar process was performed obliquely, to better position the magnets.

**Figure 4:**
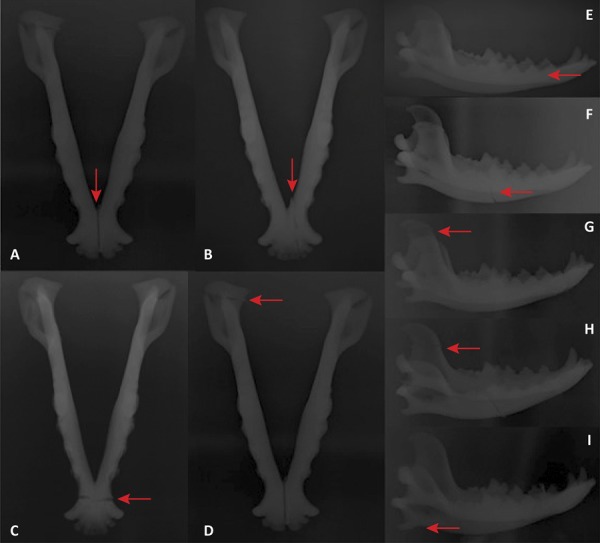
- Radiography of 3D CJF models. A. Mandibular symphysis; B. Mandibular parasymphysis; C. Canine region; D. Condylar process; E. Premolar region; F. Molar region; G. Coronoid process; H. Ramus of the mandible; I. Angular process. Arrows indicate fracture foci.

From radiographical imagery, it was possible to identify the anatomical structures corresponding to the mandible: canine tooth crowns, premolar, molar and incisor teeth, body of the mandible, condylar process, angular process, coronoid process, masseteric fossa and ramus of the mandible (Fig. 4).

Radiographs revealed radiopaque and radiolucent zones, as well as foci representing the different anatomical positions of the fractures. The images were compatible with the intended 3D model fractures.

Dorsoventral radiographs showed the fracture foci more clearly than lateral-lateral radiographs, due to the overlap of structures (right and left mandible bodies), type of filament used (ABS) and printing characteristics (fill, layer height, filament thickness, print speed and temperature).

## Discussion

The creation of 3D CJF models was based on the need to represent mandibular fractures in dogs, as this condition is clinically relevant in veterinary medicine, but is not sufficiently addressed in theoretical and practical training^17^. This may be related to the limitation of educational materials or the failure of teaching institutions in prioritizing and enforcing education in this field^20^.

The mandibular bone scan was the initial stage of the 3D CJF model production process. This method allowed for all anatomical reference points of the canine mandible to be preserved in the base model. A similar result was achieved by^9^, who scanned three bovine bones (rib, femur and cervical vertebra) to produce 3D models comparable to real bones.

Although we achieved good anatomical representation of the canine mandible in our base model, we also observed structures that were not sufficiently reproduced to serve as anatomical references. Among them was the poor representation of incisor teeth, where it was not possible to reproduce the interdental spaces. This limitation in mandibular bone scanning was also found by^21^, when reproducing a 3D model of mandibular fracture for surgical planning in human.

Another limitation of the base model was non-visualization of the mandibular canal. This limitation is directly related to the method of obtaining the images, since the 3D scanner captures only the surfaces of the mandible^3,22,23^.

For reproducing the mandibular canal and other internal anatomical structures, we could have used computerized tomography (CT) as recommended by^11,24^. When comparing the financial investment required for the acquisition and maintenance of a CT scanner, compared with that for a 3D scanner, it was evident that the 3D scanner offers greater economic viability^25^, despite these limitations.

The slightly reduced precision of the mandibular symphysis was another limitation found in the printed base model, since in the digital file the contours were well evidenced. These findings are similar to those reported by Li *et al*.^9^, where it was possible to visualize the nutrient foramina of bovine bones (femur and cervical vertebra) in a digital file, but these structures were not reliably reproduced when printed.

The low visualization of the previously mentioned structures, such as incisor interdental spaces, mandibular canal and the mandibular symphysis, does not affect the viability of the 3D CJF model. These findings corroborate the research of Thomas *et al.* ^12^, who reproduced animal skeletons, but lost some foramina and bone details. However, the absence of these details did not detract from the proposal to teach anatomy through 3D impressions.

The scanning time for creation of the base model was considered short, at 5 min. Although we did not find previous reports that described the duration of bone structure scanning, we believe that factors such as the quality of the 3D scanner used, the size of the digital image, and the low complexity of the anatomical structures in the mandible influenced the rapid scanning observed in our study.

For the modeling stage (from imagery to creation of the 3D CJF model), approximately 3 hours was required. This was much lower when compared to the modeling time for the reproduction of a canine brain, described by Schoenfeld-Tacher *et al.* ^26^. This can be explained by the complexity of the model, since it was necessary to accentuate the grooves and folds of the brain, demanding greater virtual modeling, and consequently a longer working time.

It is important to highlight that the creation time reported in our research was the time taken for the actual 3D CJF fabrication process. This value does not account for the time taken to master the 3D modeling software, print the base model and plan the process. The criterion used for assessing creation time in the present study was similar to other studies that did not consider these factors not take these factors^7,9,26^.

In order for the 3D CJF models to be feasible for the teaching, manipulation and demonstration of fractures, we chose to use neodymium magnets to detach parts of the models, allowing visualization of the fracture foci surfaces. This method of articulation is similar to that used by Preece *et al.* ^7^, who reported that the presence of magnets in 3D models allowed separation of the pieces, and visualization of the origin, and the path and insertion of ligaments and tendons of muscles, present in the distal part of the equine limb.

The time taken to print all 3D CJF models was relatively short compared with the 24 h taken to print one canine mandible, aimed at surgical planning, reported by Winer *et al.* ^11^. This sharp difference in duration can be explained by the type of material used (liquid photopolymer) and equipment used for printing (3D printer) in our study.

To compare the total cost of our models with other studies, we took into account the use of the same printing technique and similar materials. In the study by Li *et al*.^9^, 140.4 g of thermoplastic filament (PLA) was used for printing a bovine femur and the reported cost was US$3.50. The material used in the present study was also a thermoplastic filament (ABS), and used 150.1 gr of filaments, at a total cost of US$5.83. By comparison, even though printing different parts, we observed similarities in the type of material, amount used and cost of printing.

The use of 3D printed models has the potential to provide a source of high-quality teaching material^27^. However, for these educational models to be scientifically valid, methods that objectively prove their quality are needed, and should not be based only on the perception of the users^7^. Keeping it in mind, we choose to perform 3D CJF model radiographs.

For 3D CJF model imaging, two radiographic placements per model (dorsoventral and/or lateral-lateral) were sufficient. These findings are in agreement with the study by Gaia *et al*.^28^. When analyzing radiographic images of canine mandibular fractures, they found that only two radiographic positions were sufficient to indicate a fracture focus.

The radiopacity and radiolucency of the 3D CJF models did not necessarily correspond to the bone densities found in the radiography of the natural canine mandible*.* This fact can be explained by the techniques used to obtain the images (3D scanner) and manufacture the models, the material used for printing (thermoplastic), and the printing parameters used.

Another factor that may have influenced the image results is the radiographic technique used. Since the thermoplastic material is unconventional, the radiation intensity and exposure time were adjusted manually, as there are no recommended values for this material.

It was necessary to remove the neodymium magnets in the models for radiographic imaging, since in an initial test these caused visual artifacts and interfered with visualization of the fracture foci. Similar findings were reported by Panta and Yaga^29^, who analyzed X-ray and CT images of the mandible and maxilla and found that metallic artifacts limited the image quality and promoted hardened bundles that cause periodontal changes.

All radiographic images of the mandibular bone corresponded to those of the 3D CJF models. However, if these models were to be used for surgical training or for demonstration of bone diseases, they would not be representative. Surgical training requires knowledge of bone density to stabilize fractures, by means of plates and screws^11^, and for treating bone diseases we must understand the extent of the disease, the patterns of filling and the method of obtaining these images^30^.

No reports that have obtained canine mandibular radiographic images from 3D anatomical models have been found to date. Therefore, we propose the use of 3D CJF models for making radiographic images, to provide a valuable resource for the demonstration of radiographic aspects that are not usually covered in undergraduate studies.

It is important to emphasize that the methodology used in this study differs from the methodologies advocated by other researchers. The images for 3D CJF model production originated from the scanning of a natural bone, and production of the medical images (radiography) was carried out subsequently. Other studies have used the reverse approach, in that the 3D models were produced from medical images^8,25^.

Digital files generated from 3D CJF models can contribute to 3D educational model databases, so that any person or educational institution is able to access to these models^3^. An example of this possibility is the model generated by Nibblett *et al.* ^13^ which comprises the dog ear canal, and is available on the Thingiverse^©^ digital platform.

The 3D CJF models demonstrate how canine mandibular fractures can be represented and used for veterinary medicine teaching. The models have potential for use mainly in the discipline of anatomy, and the radiographic images can assist in diagnostic classes.

## Conclusions

The 3D CJF models reproduced all types of mandibular fracture that we intended to represent, as well as producing reliable radiographic images that mimicked the general anatomical aspects of the canine mandible. The 3D CJF models, and their respective radiographic images, are a possible alternative source of educational material for the teaching of veterinary medicine.

## Footnotes

1 Research performed at Laboratory of 3D Educational Technologies and Imaging Diagnostic, Center of Veterinary Medicine Teaching and Research Unit, Rio Branco-AC, Brazil.
